# Can the Reduction of Cytokines Stop the Progression of Sepsis?

**DOI:** 10.7759/cureus.22325

**Published:** 2022-02-17

**Authors:** Suna Koc, Serdar Celebi, Ferhat Hanikoglu, Yalcin Polat, Betul Borku Uysal, Mehmet Dokur, Turkan Ozer, Serap Yavuzer, Mehmet Sami Islamoglu, Mahir Cengiz, Gokay Vardar, İlke Kupeli

**Affiliations:** 1 Department of Anesthesiology and Intensive Care, Biruni University Faculty of Medicine, Istanbul, TUR; 2 Clinic of Anesthesiology and Intensive Care, Medistate Hospital, Istanbul, TUR; 3 Department of Biochemistry, Alaaddin Keykubat University, Antalya, TUR; 4 Department of Pathology, Biruni University Faculty of Medicine, Istanbul, TUR; 5 Department of Internal Medicine, Biruni University Faculty of Medicine, Istanbul, TUR; 6 Department of Emergency Medicine, Biruni University Faculty of Medicine, Istanbul, TUR; 7 Anesthesiology and Reanimation, Beysehir State Hospital, Konya, TUR; 8 Department of Medical Laboratory Techniques, Istanbul Health and Technology University, Istanbul, TUR; 9 Anesthesiology and Reanimation, Biruni University Faculty of Medicine, Istanbul, TUR

**Keywords:** intensive care, interleukin, mortality, cytokine hemadsorption, sepsis

## Abstract

Objective

In this study, we aimed to analyze the laboratory and clinical results of cytokine hemadsorption as an immunomodulation therapy in ICU patients diagnosed with sepsis or septic shock.

Methods

The levels of procalcitonin (PCT) and C-reactive protein (CRP), determined to be indicators of infection/sepsis, and the levels of interleukins (IL-6, IL-8, and IL-10) and tumor necrosis factor α (TNFα), deemed as indicators of the cytokine storm, were compared among 32 patients before and after the hemadsorption procedure.

Results

The hemadsorption significantly reduced the levels of IL-6, IL-8, IL-10, TNFα, PCT, CRP, Acute Physiology and Chronic Health Evaluation (APACHE) scores, mortality rate, and Sequential Organ Failure Assessment (SOFA) scores (p<0.05). APACHE scores and the mean predicted mortality rate (PMR) of the non-survivors measured before the procedure was significantly higher than those of survivors (p=0.002 for both). IL-10, APACHE scores, and the mortality rates determined before the hemadsorption procedure were deemed significant parameters to predict the mortality among all ICU patients (p<0.05). IL-10 levels ≤125.3 ng/L, APACHE score >30, and PMR >70.33 were significantly associated with the mortality rates of all patients, indicating that these three parameters determined before the hemadsorption may be good predictors of mortality among ICU patients with sepsis.

Conclusion

The progression of sepsis in ICU patients may be prevented with cytokine hemadsorption applied as an immunomodulator therapy.

## Introduction

Sepsis is a serious and life-threatening organ dysfunction that results from an altered regulation in the host response against infections. Sepsis is associated with high mortality and morbidity rates, reportedly between 30-50% or even higher among patients admitted in ICUs [1,2]. In Turkey, the mortality rates related to sepsis and septic shock in ICUs were reported to be 22% and 78%, respectively [3]. Sepsis triggers the stimulation of the factors in the complement system through the secretion of inflammatory cytokines including several interleukins (IL), tumor necrosis factor α (TNFα), and nitric oxide, resulting in a systemic inflammatory response [4]. This secretion of inflammatory cytokines reduces systemic vascular resistance, leading to acute hypotension and hyperlactatemia, and subsequent septic shock [5]. The combination of hypotension with microvascular obstruction leads to severe tissue ischemia, eventually resulting in multiple organ failure [2,4].

Some potential treatment mechanisms have been developed to eliminate the pro-inflammatory and anti-inflammatory cytokines in sepsis, which are positively correlated with high mortality rates [2,5,6]. One of these treatments is extracorporeal cytokine hemadsorption, which helps to reduce the excessive inflammatory response in sepsis and facilitates immunomodulation in ICU patients [1,6]. A treatment using adsorption of cytokines has been previously reported with respect to sepsis [2,4,7], but the effects of cytokine hemadsorption on ICU patients diagnosed with sepsis, as well as the progression and outcomes of the disease, were not elucidated. In this prospective study, we intended to determine the impact of cytokine hemadsorption on the clinical progression and mortality among patients diagnosed with sepsis or septic shock and admitted to the ICU.

## Materials and methods

Study design

In this single-center study, we prospectively analyzed the outcomes and laboratory findings of 39 patients diagnosed with sepsis from January 1, 2020, to December 30, 2020, who were admitted to the ICU. The inclusion criteria for the selection of patients were the availability of the data pertaining to the clinical and laboratory diagnosis of sepsis and septic shock according to “The Third International Consensus Definitions for Sepsis and Septic Shock” [8], and detection of Gram-negative bacterial agents in blood or sputum culture. Of the patients, we included those who had shock symptoms such as hypotension, tachycardia, or fever during the progression of sepsis. The exclusion criteria were uncontrolled hemorrhage, diagnosis with cardiac failure at stage 4, renal failure at stage 4, hepatic liver failure at stage 4, end-stage cancer, or admission with acute coronary syndrome.

Seven patients died due to septic shock before the end of the study and were hence excluded from the study. The remaining 32 patients diagnosed with sepsis or septic shock, hospitalized in the ICU, and undergoing a cytokine storm were selected for the study. At the end of the 90-day follow-up, all patients selected for the study were classified as either survivors or non-survivors.

The medical indications for extracorporeal cytokine hemadsorption included cytokine storm, cytokine release syndrome, hyperkalemia, acidosis, multiple organ dysfunction, or severe sepsis. The study protocol was approved by the Non-interventional Clinical Research Ethics Committee of Biruni University (No: 2021/47-28; date: January 29, 2020).

Data collection

All data including the demographics, clinical course, and laboratory findings were collected from the patients’ records in the hospital and compared between survivors and non-survivors. Inflammatory cytokines including TNFα, IL-6, IL-8, and IL-10 were dosed in a single serum sample by using flow cytometry (HA-330 disposable perfusion cartridge, Jafron Biomedical, Zhuhai, China). Hemadsorption was started on the day when the need for intubation developed and mechanical ventilation was started in patients without sepsis, and when the agent was detected in patients with sepsis. The procedure was initiated by placing a double-lumen catheter through the femoral vein or the subclavian vein. All data for hemadsorption therapy before the procedure (pre-HAD), during the procedure (intra-HAD), and after the procedure (post-HAD) were obtained. Procalcitonin (PCT) was measured by a fast test kit using the immunofluorescence assay (IA) method on a Getein 1600 device (Getein Biotech, Nanjing, China). All laboratory examinations were carried out by experienced physicians.

Survival outcomes

Survival rates of patients were determined by the length of hospitalization in the ICU or until their discharge from the hospital or exitus. The total duration of hospitalization in ICU (measured in days) was recorded before, during, and after hemadsorption treatment. The mean predicted mortality rate (PMR) of the study population was calculated based on the Acute Physiology and Chronic Health Evaluation (APACHE) scores.

Statistical analysis

Statistical analyzes were performed with the SPSS Statistics software version 23 (IBM, Armonk, NY) and MedCalc software version 18.5 (MedCalc Software Ltd, Ostend, Belgium). Continuous variables were presented as means and standard deviation, minimum, maximum, and median values, and categorical variables as frequencies and percentages. The normality of distribution was tested with Shapiro Wilk's Lambda test. The independent variables were analyzed by the Mann-Whitney U test; two dependent variables were analyzed by the Wilcoxon signed-ranks test, and three or more dependent variables were analyzed by the Friedman test. The variables that were significant in the prediction of mortality were analyzed by the receiver operating characteristic (ROC) curve. The statistical significance was set at p<0.05.

## Results

The demographics of 32 patients diagnosed with sepsis are presented in Table 1; 26 patients (81.25%) died during the 90-day follow-up. No significant difference was found regarding gender between survivors and non-survivors; however, the median age of the non-survivor group was significantly higher than that of the survivor group (p=0.026).

**Table 1:** Demographics of all patients with sepsis

		Survivors (n=6)	Non-survivors (n=26)	P-value
Gender, n (%)	Female	4 (26.67)	11 (73.33)	0.383
	Male	2 (11.76)	15 (88.24)	
Age, years	Median (range)	52.0 (42.0–75.0)	74.5 (25.0–96.0)	0.026

The laboratory parameters measured before, during, and after the extracorporeal hemadsorption procedure were compared among all patients with sepsis and the outcomes are presented in Table 2. The mean levels of TNF-α, IL-6, IL-10, PCT, and CRP measured during and after the hemadsorption were significantly lower compared to the levels measured before the procedure (p<0.05). The mean level of IL-8, the median of APACHE score, the PMR based on APACHE score, and SOFA score determined after the procedure were significantly lower compared to those determined before the procedure for all patients (p<0.05).

**Table 2:** Comparison of the laboratory parameters of patients according to the periods of extracorporeal hemadsorption ^a^P<0.05 vs pre-HAD IL-6. ^b^P<0.05 vs pre-HAD IL-8. ^c^P<0.05 vs pre-HAD IL-10. ^d^P<0.05 vs pre-HAD TNF-α. ^e^P<0.05 vs pre-HAD PCT. ^f^P<0.05 vs pre-HAD CRP SD: standard deviation; HAD: hemadsorption; IL: interleukin; TNFα: tumor necrosis factor α; PCT: procalcitonin; CRP: C-reactive protein; APACHE: Acute Physiology and Chronic Health Evaluation; PMR: predicted mortality rate of the study population calculated based on APACHE score; SOFA: Sequential Organ Failure Assessment

Parameter	Period	Mean ± SD; median (range)	P-value
IL-6 (ng/L)	Pre-HAD	129.32 ± 41.24; 115.05 (54.3–225.8)	0.001
	Intra-HAD	101.7 ± 36.3^a^; 89.1 (47.2–177.4)	
	Post-HAD	88.35 ± 55.01^a^; 84.7 (5.7–206.4)	
IL-8 (ng/L)	Pre-HAD	86.14 ± 58.34; 81.85 (7.87–215.4)	0.0001
	Intra-HAD	52.76 ± 47.98; 45.46 (0.14–158.2)	
	Post-HAD	38.37 ± 40.36^b^; 24.51 (1.24–154.3)	
IL-10 (ng/L)	Pre-HAD	172.33 ± 187.72; 117.2 (23.15–856.3)	0.0001
	Intra-HAD	115.77 ± 88.89^c^; 93.95 (14.9–389.2)	
	Post-HAD	82.8 ± 61.34^c^; 69.7 (0.15–292.2)	
TNFα (pg/mL)	Pre-HAD	86.85 ± 49.9; 71.65 (12.45–212.2)	0.0001
	Intra-HAD	52.61 ± 34.44^d^; 48.5 (3.24–151.5)	
	Post-HAD	22.64 ± 20.37^d^; 14.6 (1.23–98.2)	
PCT (ng/mL)	Pre-HAD	6.74 ± 11.09; 2.17 (0.28–57)	0.001
	Intra-HAD	2.24 ± 3^e^; 0.95 (0.1–15)	
	Post-HAD	2.2 ± 3.15^e^; 0.61 (0.06–4)	
CRP (mg/mL)	Pre-HAD	183.75 ± 70; 176 (63–350)	0.0001
	Intra-HAD	121.38 ± 57.71^f^; 108.5 (37–259)	
	Post-HAD	97.57 ± 70.21^f^; 83 (9.2–338)	
APACHE score	Pre-HAD	33.94 ± 9.71; 35.5 (13–50)	0.001
	Post-HAD	26.94 ± 9.47; 26.5 (8–47)	
PMR	Pre-HAD	73.76 ± 22.81; 80.02 (16.54–98.01)	0.001
	Post-HAD	58.94 ± 21.99; 55.47 (11.34–96.53)	
SOFA score	Pre-HAD	16.06 ± 3.08; 16 (10–24)	0.0001
	Post-HAD	12.48 ± 3.79; 12 (5–21)	

The laboratory parameters measured before the hemadsorption were compared between the groups and the outcomes are presented in Table 3. The mean levels of TNFα, IL-6, IL-8, IL-10, PCT, CRP, and SOFA score did not differ between the non-survivor and survivor groups (p>0.05). However, the median APACHE score of non-survivors was significantly greater than that of survivors (p=0.002). Moreover, the mean PMR of the study population determined before the procedure was found to be elevated among the non-survivors compared with that of the survivors (p=0.002).

**Table 3:** Comparison of the laboratory parameters of the patients recorded before the extracorporeal hemadsorption SD: standard deviation; IL: interleukin; TNFα: tumor necrosis factor α; PCT: procalcitonin; CRP: C-reactive protein; APACHE: Acute Physiology and Chronic Health Evaluation; PMR: predicted mortality rate of the study population calculated based on APACHE score; SOFA: Sequential Organ Failure Assessment

Parameter	Survivors (n=6), mean ± SD; median (range)	Non-survivors (n=26), mean ± SD; median (range)	P-value
IL-6 (ng/L)	125.48 ± 29.55; 115.05 (95.6–174.3)	130.2 ± 43.93; 114.95 (54.3–225.8)	0.961
IL-8 (ng/L)	66.68 ± 41.32; 67.71 (20.23–120.01)	90.63 ± 61.37; 88.35 (7.87–215.4)	0.469
IL-10 (ng/L)	296.55 ± 292.33; 180.85 (58.3–856.3)	143.66 ± 148.48; 115.25 (23.15–678.1)	0.06
TNFα (pg/mL)	81.18 ± 58.25; 61.8 (33.69–196.2)	88.16 ± 48.98; 76.2 (12.45–212.2)	0.53
PCT (ng/mL)	8.22 ± 10.77; 1.81 (0.3–22.2)	6.4 ± 11.34; 2.17 (0.28–57)	0.923
CRP (mg/mL)	173.83 ± 90.48; 183.5 (63–312)	186.04 ± 66.41; 176 (100–350)	0.664
APACHE score	22 ± 7.24; 22 (13–30)	36.69 ± 8.02; 37.5 (18–50)	0.002
PMR	44.37 ± 23.94; 44.37 (16.54–70.33)	80.54 ± 16.6; 86.81 (29.13–98.01)	0.002
SOFA score	15.33 ± 0.82; 15.5 (14–16)	16.23 ± 3.39; 16 (10–24)	0.624

**Figure 1:**
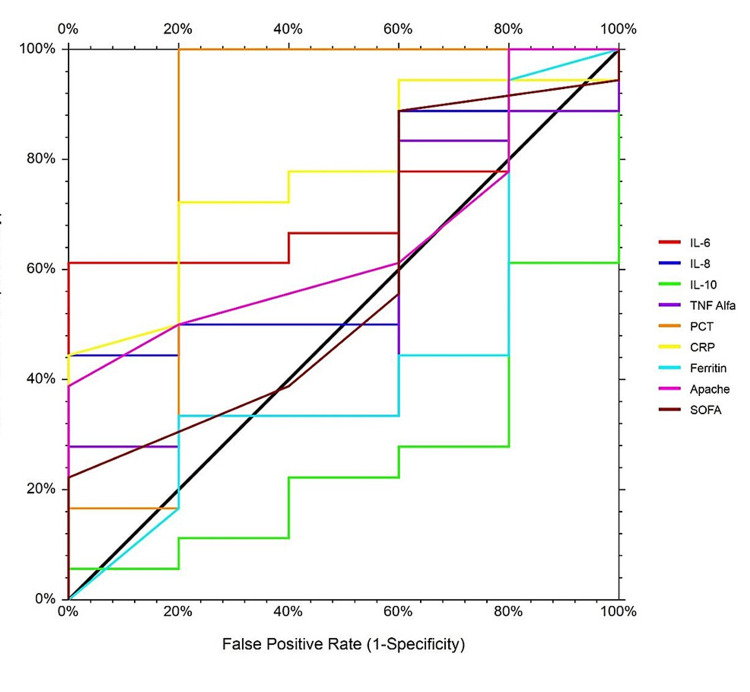
Laboratory ROC analysis before hemadsorption to predict mortality ROC: receiver operating characteristic; IL: interleukin; TNFα: tumor necrosis factor α; PCT: procalcitonin; CRP: C-reactive protein; APACHE: Acute Physiology and Chronic Health Evaluation; SOFA: Sequential Organ Failure Assessment

The receiver operating characteristic (ROC) curve analysis of pre-hemadsorption laboratory parameters in predicting the mortality showed that IL-10, APACHE score, and PMR determined before the hemadsorption procedure were significant parameters to predict the mortality among all ICU patients (p<0.05; Table 4). IL-10 levels ≤125.3 ng/L [area under the ROC curve (AUC): 0.75, 95% CI%: 0.566-0.885], APACHE score >30 (AUC: 0.907, 95% CI%: 0.751-0.981), and PMR >70.33 (AUC: 0.907, 95% CI%: 0.751-0.981) determined before the procedure were significantly associated with PMRs of all patients (Figure 1).

**Table 4:** ROC curve analysis of pre-hemadsorption laboratory parameters in predicting the mortality among the patients ROC: receiver operating characteristic; HAD: hemadsorption; PPV: positive predictive value; NPV: negative predictive value; IL: interleukin; APACHE: Acute Physiology and Chronic Health Evaluation; PMR: predicted mortality rate of the study population calculated based on APACHE score

Pre-HAD parameters	Cut-off	Sensitivity (95% CI)	Specificity (95% CI)	PPV (95% CI)	NPV (95% CI)	AUC (95% CI)	P-value
IL-10	≤125.3	73.08 (52.2–88.4)	83.33 (35.9–99.6)	95 (75.8–99.1)	41.7 (25.7–59.7)	0.75 (0.566–0.885)	0.0406
APACHE score	>30	73.08 (52.2–88.4)	100 (54.1–100.0)	0	46.2 (31.3–61.8)	0.907 (0.751–0.981)	<0.0001
PMR	>70.33	69.23 (48.2–85.7)	100 (54.1–100.0)	0	42.9 (29.6–57.2)	0.907 (0.751–0.981)	<0.0001

## Discussion

Currently, there are various adjuvant therapy modalities for the management of excessive cytokine production in sepsis, including immunoglobulin therapy, plasma filtration, endotoxin-binding polymyxin B, and hemoperfusion. However, these techniques carry a high risk of nephrotoxicity as well as acute blood loss in patients hospitalized in ICUs [9,10,11]. One alternative treatment to these modalities is the extracorporeal cytokine hemadsorption, which is currently used in clinical practice as a safe and well-tolerated technique to reduce significant serum cytokine concentrations. In the present study, we investigated the effectiveness of this method on the clinical progression and mortality among 32 ICU patients diagnosed with sepsis or septic shock. It was found that the mean levels of TNFα, IL-6, IL-10, PCT, and CRP measured during and after the extracorporeal cytokine hemadsorption significantly decreased compared to the baseline levels. The mean level of IL-8, the median APACHE score, the mortality rate, and SOFA scores determined following the procedure were found significantly reduced compared to those determined before the procedure for all patients; 26 patients (81.25%) died during the 90-day follow-up.

An extracorporeal cytokine adsorption device (CytoSorb®, CytoSorbents Corporation, Monmouth Junction, NJ) used with current standard care including mechanical ventilation and dialysis has been shown to reduce the pro-inflammatory cytokines, resulting in regulated hemodynamics in sepsis and septic shock [12]. This device has been reported to provide a safe and well-tolerated therapy in ICU patients in the advanced stage of sepsis and lung injury [13]. Recently, routine care in the management of sepsis and septic shock with the aid of CytoSorb® therapy has been proven to improve clinical outcomes. This system has been shown to decrease the mortality rates of patients with septic shock, improve hemodynamics by regulating the mean arterial pressure, and reduce the use of vasopressors and their doses. In addition, the therapy achieved significantly lower APACHE II and SOFA scores, especially in the survivor group (APACHE II score: 20.1 ± 2.47 and SOFA score: 9.04 ± 3.00) [14]. These scores significantly decreased after the cytokine hemadsorption therapy in our study as well. Our findings are consistent with the outcomes reported in the literature [15,16].

Cytokines play a major role in the pathophysiology of sepsis. Increased levels of pro-inflammatory cytokines in the serum result in a disturbance in the immune system, inducing multi-organ failure that may cause prolonged hospitalization and elevated mortality rates among ICU patients [17,18]. In the present study, several cytokines were examined in the 32 ICU patients having sepsis or septic shock whose indications included cytokine storm, cytokine release syndrome, hyperkalemia, acidosis, and multiple organ dysfunction. The levels of cytokines before the cytokine hemadsorption were compared in both the survivor and non-survivor groups. The median age and APACHE score of non-survivors were notably higher than those of survivors. However, the mean levels of TNF-α, IL-6, IL-8, IL-10, PCT, CRP, and SOFA score did not differ between the two groups. The ROC curve analysis of these parameters revealed that IL-10 levels and APACHE score determined before the procedure were significant parameters to predict mortality among all ICU patients. In addition, the cut-off levels of IL-10 ≤125.3 were ng/L (AUC: 0.75, 95% CI%: 0.566-0.885), and those of APACHE score were >30 (AUC: 0.907, 95% CI%: 0.751-0.981), suggesting an association with the mortality rates of all patients. In a study by Singbartl et al., the elevated serum levels of IL-1 and IL-6 were found to be correlated with the severity of sepsis and resultant organ damage [19,20]. In a recent prospective study, Paul et al. used the CytoSorb® device to perform extracorporeal cytokine adsorption in 45 patients with sepsis and septic shock who were hospitalized in the ICU and reported elevated levels of IL-6 in the survivor group of patients [15]. However, we did not find any difference in the mean IL-6 levels between survivors and non-survivors in our study, probably due to the small sample size and different clinical indications of patients. Paul et al. also found the mean APACHE II score and SOFA score measured before applying the adsorption therapy in the survivor group to be 25.46 ± 2.91 and 12.90 ± 4.02, respectively [14]. In our study, the APACHE score determined before the procedure was lower (22 ± 7.24) while the SOFA score was higher (15.33 ± 0.82) in survivors compared to the findings of Paul et al. Moreover, non-survivors showed significantly higher APACHE scores than those of survivors, suggesting that this score combined with the IL levels may be efficient in predicting the mortality among patients diagnosed with sepsis or septic shock in ICUs. These findings of our study are partially supported by the above-mentioned studies, indicating that it is vital to examine biochemical parameters in detail.

The PMR calculated based on APACHE scores can be employed in determining the functioning of an ICU and to compare different ICUs in terms of the clinical progression of patients. In a study by Venkataraman et al., the reported mean PMR of patients hospitalized in the ICU was 44.8 ± 26.7 according to the APACHE II scoring system and 29.1 ± 28.5 according to the APACHE IV system. The overall mortality rate was found to be 22.4% [21]. In another study, 26 critically ill patients with septic shock were treated with CytoSorb® therapy, and the rates of 28-day mortality, mortality in ICU, and mortality in the hospital were 61.54%, 73.08%, and 80.77%, respectively. However, the mortality predicted by the APACHE II score was 89.9%, which was higher than the overall mortality in the cohort [22]. Another study evaluating hemadsorption using CytoSorb® reported that the observed vs. expected 28-day mortality decreased in patients with septic shock, and the mean PMR calculated by SOFA was 75% while the actual mortality was found to be 48% [23]. In a very recent study, Paul et al. calculated PMR before CytoSorb® therapy as 56.5% in the ICU patients diagnosed with sepsis and septic shock, while the actual mortality following the CytoSorb® therapy was determined to be 48.8% [14]. In the present study, PMR determined before the hemadsorption procedure (73.76 ± 22.81) was a significant parameter to predict the mortality among all ICU patients; it decreased after the procedure to 58.94 ± 21.99, which was consistent with the literature. In addition, the actual mortality rate was 81.25% after a 90-day follow-up. The mean PMR of the patients determined before the procedure was higher among non-survivors compared with the rates of survivors (80.54 ± 16.6 vs. 44.37 ± 23.94, respectively).

This study has some limitations. It had a relatively small sample size and unstandardized demographics including the age difference between survivors and non-survivors. Moreover, there was no control group to assess the efficiency of cytokine hemadsorption. Also, detailed hemodynamic parameters were not measured. There was also no safety data showing the possible complications of the procedure including thrombocytopenia. However, a very recent prospective study has provided the safety data for the therapy as the platelet count remained stable in it [24].

## Conclusions

Overall, the current study showed reductions in cytokine levels, CRP, and PCT levels among ICU patients diagnosed with sepsis or septic shock following cytokine hemadsorption therapy. Our findings support the concept that IL-10 level and APACHE score determined before the procedure correlated with the mortality rates of septic patients. Hence, the extracorporeal cytokine hemadsorption might be an efficient adjuvant therapy to reduce circulating cytokine levels and regulate the hemodynamics of septic shock patients. However, further multicenter randomized case-control studies are required to validate our findings and establish this therapy as a standard method for use in clinics. Moreover, future clinical trials are required to evaluate other cytokines and hematological parameters in critically ill patients diagnosed with sepsis and undergoing cytokine hemadsorption therapy.

## References

[1] Angus DC, van der Poll T (2013). Severe sepsis and septic shock. N Engl J Med.

[2] Houschyar KS, Pyles MN, Rein S (2017). Continuous hemoadsorption with a cytokine adsorber during sepsis - a review of the literature. Int J Artif Organs.

[3] Bahar İ, Oksuz H, Şenoğlu N (2021). Compliance with the surviving sepsis campaign bundle: a multicenter study from Turkey. Cureus.

[4] László I, Trásy D, Molnár Z, Fazakas J (2015). Sepsis: from pathophysiology to individualized patient care. J Immunol Res.

[5] Hotchkiss RS, Moldawer LL, Opal SM, Reinhart K, Turnbull IR, Vincent JL (2016). Sepsis and septic shock. Nat Rev Dis Primers.

[6] Rhodes A, Evans LE, Alhazzani W (2017). Surviving Sepsis Campaign: International Guidelines for Management of Sepsis and Septic Shock: 2016. Intensive Care Med.

[7] Rhodes A, Evans LE, Alhazzani W (2017). Surviving Sepsis Campaign: International Guidelines for Management of Sepsis and Septic Shock: 2016. Crit Care Med.

[8] Ye Q, Wang B, Mao J (2020). The pathogenesis and treatment of the `Cytokine Storm' in COVID-19. J Infect.

[9] Singer M, Deutschman CS, Seymour CW (2016). The Third International Consensus definitions for sepsis and septic shock (Sepsis-3). JAMA.

[10] Cruz DN, Antonelli M, Fumagalli R (2009). Early use of polymyxin B hemoperfusion in abdominal septic shock: the EUPHAS randomized controlled trial. JAMA.

[11] Kreymann KG, de Heer G, Nierhaus A, Kluge S (2007). Use of polyclonal immunoglobulins as adjunctive therapy for sepsis or septic shock. Crit Care Med.

[12] Bellomo R, Baldwin I, Ronco C (2001). Extracorporeal blood purification therapy for sepsis and systemic inflammation: its biological rationale. Contrib Nephrol.

[13] Bonavia A, Groff A, Karamchandani K, Singbartl K (2018). Clinical utility of extracorporeal cytokine hemoadsorption therapy: a literature review. Blood Purif.

[14] Reshma Tewari DRBG (2022). Reshma Tewari: role of Cytosorb® in optimization of vasopressors and reduction of sepsis scores: a case series. https://cytosorb-therapy.com/wpcontent/uploads/2015/10/Tewari_Poster_2015_10_02_CytoSorb.pdf.

[15] Paul R, Sathe P, Kumar S, Prasad S, Aleem M, Sakhalvalkar P (2021). Multicentered prospective investigator initiated study to evaluate the clinical outcomes with extracorporeal cytokine adsorption device (CytoSorb®) in patients with sepsis and septic shock. World J Crit Care Med.

[16] Hawchar F, László I, Öveges N, Trásy D, Ondrik Z, Molnar Z (2019). Extracorporeal cytokine adsorption in septic shock: a proof of concept randomized, controlled pilot study. J Crit Care.

[17] Träger K, Skrabal C, Fischer G (2017). Hemoadsorption treatment of patients with acute infective endocarditis during surgery with cardiopulmonary bypass - a case series. Int J Artif Organs.

[18] Gouel-Chéron A, Allaouchiche B, Guignant C, Davin F, Floccard B, Monneret G (2012). Early interleukin-6 and slope of monocyte human leukocyte antigen-DR: a powerful association to predict the development of sepsis after major trauma. PLoS One.

[19] Mera S, Tatulescu D, Cismaru C (2011). Multiplex cytokine profiling in patients with sepsis. APMIS.

[20] Singbartl K, Miller L, Ruiz-Velasco V, Kellum JA (2016). Reversal of acute kidney injury-induced neutrophil dysfunction: a critical role for resistin. Crit Care Med.

[21] Venkataraman R, Gopichandran V, Ranganathan L, Rajagopal S, Abraham BK, Ramakrishnan N (2018). Mortality prediction using Acute Physiology and Chronic Health Evaluation II and Acute Physiology and Chronic Health Evaluation IV scoring systems: is there a difference?. Indian J Crit Care Med.

[22] Kogelmann K, Jarczak D, Scheller M, Drüner M (2017). Hemoadsorption by CytoSorb in septic patients: a case series. Crit Care.

[23] Brouwer WP, Duran S, Kuijper M, Ince C (2019). Hemoadsorption with CytoSorb shows a decreased observed versus expected 28-day all-cause mortality in ICU patients with septic shock: a propensity-score-weighted retrospective study. Crit Care.

[24] Barsam SJ, Psaila B, Forestier M (2011). Platelet production and platelet destruction: assessing mechanisms of treatment effect in immune thrombocytopenia. Blood.

